# Environmental Health Disparities: A Framework Integrating Psychosocial and Environmental Concepts

**DOI:** 10.1289/ehp.7074

**Published:** 2004-08-16

**Authors:** Gilbert C. Gee, Devon C. Payne-Sturges

**Affiliations:** ^1^University of Michigan School of Public Health, Department of Health Behavior and Health Education, Ann Arbor, Michigan, USA; ^2^Office of Policy, Economics and Innovation, and Office of Children’s Health Protection, U.S. Environmental Protection Agency, Washington, DC, USA

**Keywords:** environmental, environmental justice, ethnicity, framework, health disparities, psychosocial, race, review, stress

## Abstract

Although it is often acknowledged that social and environmental factors interact to produce racial and ethnic environmental health disparities, it is still unclear how this occurs. Despite continued controversy, the environmental justice movement has provided some insight by suggesting that disadvantaged communities face greater likelihood of exposure to ambient hazards. The exposure–disease paradigm has long suggested that differential “vulnerability” may modify the effects of toxicants on biological systems. However, relatively little work has been done to specify whether racial and ethnic minorities may have greater vulnerability than do majority populations and, further, what these vulnerabilities may be. We suggest that psychosocial stress may be the vulnerability factor that links social conditions with environmental hazards. Psychosocial stress can lead to acute and chronic changes in the functioning of body systems (e.g., immune) and also lead directly to illness. In this article we present a multidisciplinary framework integrating these ideas. We also argue that residential segregation leads to differential experiences of community stress, exposure to pollutants, and access to community resources. When not counterbalanced by resources, stressors may lead to heightened vulnerability to environmental hazards.

The elimination of disparities in environmental health requires attention to both environmental hazards and social conditions [U.S. Environmental Protection Agency (EPA) 2003a; Institute of Medicine 1999]. However, two major challenges are implicit in this statement: first, to understand how social processes may interrelate with environmental toxicants, and second, to understand why some groups experience greater illness compared with other groups. Our purpose in this article is to provide a multidisciplinary framework that addresses both issues.

We extend the work of Sexton et al. (1993), who documented how the exposure–disease paradigm could explain variation in the health of disadvantaged populations. Implicit in their framework is the idea that disadvantaged populations encounter greater susceptibility to environmental hazards. However, it is unclear what these susceptibility factors might be.

We suggest that psychosocial stress is a key component of differential susceptibility. Stressors, when not ameliorated by resources, may directly lead to health disparities. Additionally, stressors may amplify the effects of toxicants. Residential segregation may be one important reason why communities differ in these exposures (Massey and Denton 1993).

Our framework is built on an ecological perspective, suggesting that health disparities result not only from individual factors but also from factors operating at multiple levels (Bronfenbrenner 1989; Diez-Roux 1998; Pickett and Pearl 2001; Sallis and Owen 1997). Reinvigoration in ecological approaches has paralleled the development of statistical techniques of multilevel modeling (e.g., hierarchical linear models), an appreciation that ecological factors may not necessarily lead to the ecological fallacy, and a renewed interest in the role of context in health promotion (Diez-Roux 2000; Green and Kreuter 1999).

## Health Disparities and the Environment

Disparities exist for many health outcomes, including cancer, cardiovascular disease, diabetes, and mortality [U.S. Department of Health and Human Services (DHHS) 2000]. Although there has been a national decrease in disparities between 1990 and 1998 (Keppel et al. 2002), some regions have reported an increase in disparities during the same period (Margollos et al. 2004).

Environmental conditions are believed to play an important role in producing and maintaining health disparities (Lee 2002; Sexton 2000; Yen and Syme 1999). Minority neighborhoods tend to have higher rates of mortality, morbidity, and health risk factors compared with white neighborhoods, even after accounting for economic and other characteristics (Cubbin et al. 2001; Deaton and Lubotsky 2003; Geronimus et al. 2001).

## The Stress–Exposure Disease Framework

**Figure 1:**
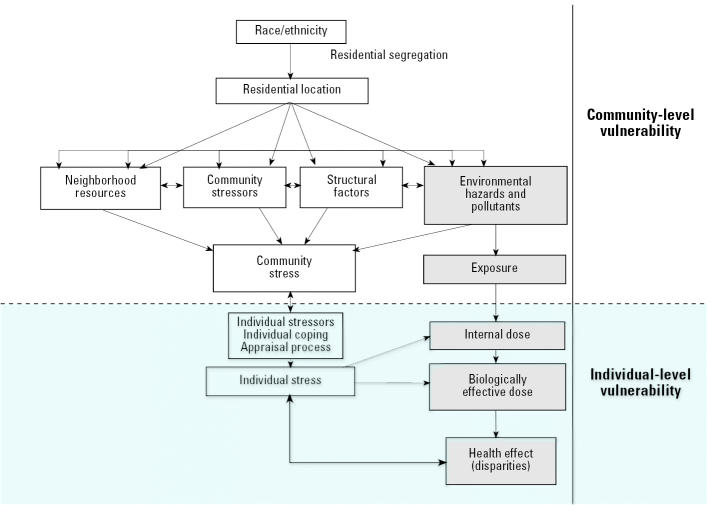
Exposure–disease–stress model for environmental health disparities.

The stress–exposure disease framework (Figure 1) provides a conceptual framework from which to understand the relationships among race, environmental conditions, and health. It extends the framework of Sexton et al. (1993) by *a*) explicitly hypothesizing that residential segregation is a major reason why “race” is important; *b*) incorporating an ecological or multilevel perspective; and *c*) arguing that racial variation in stressors may account for differences in vulnerability to health risks.

Reflecting the multilevel approach, Figure 1 emphasizes both community processes (top) and individual mechanisms (bottom). The shading reflects the exposure–disease paradigm. To simplify our presentation, we have separated individual and community processes. However, many processes are interrelated. For example, community wealth is partly a function of individual wealth (e.g., when individuals contribute to the tax base), and individual wealth is also partly determined by community wealth (e.g., when rising property values benefit individual homeowners).

The framework shows that ethnicity is highly correlated with residential location, with minorities and whites often living segregated from one another. Differential residential location comes with differential exposure to health risks. In particular, neighborhood stressors and pollution sources create adverse health conditions, which are counterbalanced by neighborhood resources. Structural factors help determine the boundaries from which health promotion is possible and partially determine the contemporary state of stressors, resources, and pollution in a community. When community stressors and pollution sources outweigh neighborhood resources, levels of community stress manifest or increase. Community stress is a state of ecological vulnerability that may translate into individual stressors, which in turn may lead to individual stress. Individual stress may then make individuals more vulnerable to illness when they are exposed to environmental hazards. Further, compromises in individual and community health may further weaken community resources, leading to a vicious cycle. Hence, we include in our framework a return loop from health back to stress.

As an example, zoning policies and tax incentives (structural factors) may encourage the entry of new pollutant industries. The increase in pollutants may lead to economic and social uncertainty (stressors) by driving down local property values, increasing the flight of jobs and fostering a climate of uncertainty and fear. Neighborhood organizations (resources) may not be able to counterbalance these effects, leading to a state of community vulnerability (community stress). Community-level vulnerability, in turn, may translate to individual vulnerability, such as when individuals lose their jobs or become anxious about perceived toxic exposures. When personal coping resources do not adequately counterbalance these external insults, individual stress and illness may result. Individual illness, in turn, may lead to further individual vulnerability, such as by reducing the ability to exercise. Additionally, individuals may affect their communities, such as when disaffected individuals cease participating in neighborhood organizations. Health disparities may arise because minorities are segregated into neighborhoods with high levels of community stress.

We do not explicitly examine the issue of genetic susceptibility in this framework for three reasons. First, we focus on factors that are amenable to policy change and social action. Second, genetic susceptibility is partly subsumed in the exposure–disease paradigm because it is presumed to partially determine one’s ability to defend against hazards. Third, although genetic factors are important in the etiology of many illnesses, it is likely that genetic factors do not explain racial health disparities (Cooper 1984; Cooper et al. 2003; Garte 2002; LaVeist 1994). It is often acknowledged that race is a social construct. What that means is that racial groups are not inherent biological taxons, but represent societally defined categories during a particular point in history and place. For example, before 1989, the child of a black father and a white mother would be classified as black, but after 1989, the same child would be classified as white (LaVeist 1994). Further, a child born in Brazil, rather than the United States, would be classified as mulatto. Thus, racial designations are the product of social consensus and public policy, rather than biology per se.

Additionally, “genetically identified” groups tend to correlate poorly with socially identified groups because there is more genetic variation within than between groups (Garte 2002; Lewontin 1982; Mountain and Cavalli-Sforza 1997). For example, genetic differences between any two Italians appear to be 5-fold greater than the difference between an Italian and a Japanese, African, or New Guinean (Mountain and Cavalli-Sforza 1997). Observations such as these have led Cooper et al. (2003) to conclude that race “has not shown to provide a useful categorization of genetic information about the response to drugs, diagnosis, or causes of disease.”

We now review the science that informs this framework, beginning with the exposure–disease paradigm.

### The Exposure–Disease Paradigm

The exposure–disease paradigm is a well-known model that shows how environmental toxicants might cause disease (Lioy 1990; Lioy and Pellizzari 1995; National Research Council, 1991a, 1991b; Wagener 1987). It is a continuum that includes the emission of a contaminant from a source through human exposure to the occurrence of a health effect.

Susceptibility/vulnerability intersects the continuum, increasing or decreasing resistance to absorption and/or effect from toxicants. The term “susceptibility/vulnerability” has been used broadly to cover both biological and non-biological factors, including genetic predisposition, pre-existing health conditions, and social conditions. The exact susceptibility/ vulnerability factors and their pathways intersecting the exposure–disease paradigm are not well understood. We argue later that community and individual stress is one type of susceptibility factor.

### Race and Residential Location

Segregation, the spatial separation of the residences of racial groups from one another, has persisted for many decades (Iceland et al. 2002; Massey 2001; Massey and Denton 1993). Table 1 shows the segregation of blacks, Hispanics, Native Americans, and Asians compared with whites from 1980 to 2000 for metropolitan areas, as measured with the index of dissimilarity (Logan 2003; U.S. Census Bureau 2003). Scored from 0 to 100, a given value of the index indicates the percentage of that group who would have to move to integrate the metropolitan area.

Segregation from whites is highest for African Americans, followed by Hispanics, Asian Americans, and Native Americans. In the average U.S. metropolis in the year 2000, about two-thirds of blacks (or whites) would have to move to another neighborhood in order to desegregate that metropolis.

Black–white and Native-American–white segregation has declined since the 1980s, but segregation levels for Hispanics and Asians have remained stable. Further, most of the decline in black–white segregation has occurred in metropolitan areas with the fewest numbers of blacks (Logan 2003).

The causes of segregation are still debated. Some have suggested that segregation is an artifact of broader shifts in the economy—including the decline of manufacturing jobs and suburbanization—that have left behind a cadre of the poor that are disproportionately racial minorities (Wilson 1987, 1996). Others have postulated that segregation results from personal preferences of homebuyers to cluster together (Schelling 1971). Most research has argued that segregation results from institutionalized discriminatory practices in the housing market (e.g., mortgage redlining, racialized “steering”) that persists to the current day (Massey and Denton 1993; Meyer 2000; Munnell et al. 1996; Schwartz 1998; Squires 1994; Squires and Velez 1996).

Some evidence suggests that the mechanisms for segregation vary by ethnic group and region, but most ethnic groups have encountered discriminatory treatment historically and currently (Squires 1994; Feagin and McKinney 2003; Krieger et al. 1993; Williams et al. 1997). For example, a recent audit study suggested that consistent adverse treatment in home buying was similar for Asian-American and African-American homebuyers, with one in five potential homebuyers disfavored compared with whites (Turner and Skidmore 2001). The causes of segregation notwithstanding, it is clear that neighborhoods do cluster on the basis of race and ethnicity.

Studies have reported that segregation is associated with numerous outcomes, including infant mortality [Centers for Disease Control and Prevention (CDC) 2002; LaVeist 1989, 1993], adult mortality (Hart et al. 1998; Jackson et al. 2000; Polednak 1991, 1996), tuberculosis (Acevedo-Garcia 2003), homicide (Peterson and Krivo 1993, 1999), teenage childbearing (Sucoff and Upchurch 1998), exposure to tobacco and alcohol advertising (Alaniz 1998; Luke et al. 2000; U.S. DHHS 1998), and increased exposure to air pollution (Lopez 2002).

Segregation may thus be one critical link between race and environmental health disparities because racial groups, on average, occupy different residential areas. This may lead to differential exposure to health risk factors as well as differential access to resources. Segregation is multifactorial, often conceptualized around five dimensions (Acevedo-Garcia 2000; Massey and Denton 1988, 1993): *a*) evenness, the inequitable distribution of groups over an area and the dimension receiving the greatest empirical study; *b*) isolation, the degree of potential contact between two groups within a city; *c*) concentration, the extent to which minority groups are confined to a compact area within the city; *d*) centralization, the degree to which minorities are clustered around the center of a city; and *e*) clustering, the extent to which minority neighborhoods are adjacent to one another. Our discussion refers to the general principle of segregation, although it will be an important research endeavor to examine which specific dimensions of segregation are related to environmental health disparities.

Having established a link between race and residence, we now turn to the proximal mechanisms that may account for the relationship between environmental conditions and racial health disparities.

### Environmental Hazards and Pollutants

Briefly, environmentally relevant disparities are evident in a variety of outcomes, including asthma, cancer, and chemical poisoning (Institute of Medicine 1999). Although debated, the main hypothesis explaining these disparities is that disadvantaged communities encounter greater exposure to environmental toxicants such as air pollution, pesticides, and lead (Burger et al. 2001; Calderon et al. 1993; Corburn 2002; Fitzgerald et al. 1998; Institute of Medicine 1999; Morello-Frosch 2001; Moses et al. 1993; Northridge et al. 2003; Perera 2003; Pirkle et al. 1998; Woodruff et al. 2003). Mediators of the relationship between toxic exposure and disadvantaged status include the siting of pollution sources (e.g., waste incinerators), illegal dumping, poor enforcement of environmental regulations, and inadequate response to community complaints (Anderton et al. 1994, 1997; Bullard 1983, 1990; Bullard and Wright 1993; Goldman and Fitton 1994; Institute of Medicine 1999; Maantay 2001, 2002; Mohai and Bryant 1992; Perlin et al. 1999, 2001; United Church of Christ 1987; U.S. General Accounting Office 1983).

### Structural Factors

Structural factors refer to the historically evolving infrastructure that provides boundaries for health promotion. That is, structural factors are constraints that shape how new conditions emerge as “salutogens” (factors that support health) or pathogens in a community. The local economy, for example, is a structural factor that will help determine a community’s ability to mobilize resources in order to reject undesirable changes (e.g., introduction of a waste facility) or develop desirable ones (e.g., construction of a park). Structural factors that may be especially pertinent to environmental health disparities include the local and national economy, neighborhood physical conditions, land use patterns, and health infrastructure. This is not an exhaustive list, but rather is meant to be illustrative.

One primary effect of residential segregation may be to concentrate disadvantage (Massey and Denton 1993). Compared with whites, minorities are overrepresented in neighborhoods with diminishing and constrained economic opportunities (Jargowsky 1997; Wilson 1987). For example, in Los Angeles, California, in 1990, only 4.9% of blacks lived in high-job-growth areas, compared with 52.3% of whites (Pastor 2001). Cutler and Glaeser (1997) reported that a decrease in segregation by one standard deviation (13%) would eliminate one-third of the black–white differences in education and employment. Thus, segregation not only may concentrate poverty but also may be partly responsible for the production of poverty among racial minorities (Massey and Denton 1993; Williams and Collins 2001).

There is a clear association between socioeconomic position and health, such that individuals of higher social standing tend to have improved health compared with those of lower standing (Evans and Kantrowitz 2002; Kaplan et al. 2001; Krieger and Fee 1994; Marmot et al. 1987, 1998; O’Neill et al. 2003; Williams and Collins 1995). Further, the relationship between socioeconomic position and health holds not only at the individual level but also at the community level (Haan et al. 1987; Kaplan 1996). That is, persons living in poor neighborhoods, even after accounting for their individual socioeconomic characteristics, tend to have worse health outcomes (Diez-Roux et al. 1997, 2001; Merkin et al. 2002; Waitzman and Smith 1998; Winkleby and Cubbin 2003).

Neighborhood economic deprivation may compromise health-promoting resources (Diez-Roux et al. 2001). For example, poor and minority neighborhoods tend to have fewer grocery stores with healthy foods (Morland et al. 2002) and fewer pharmacies with needed medications (Morrison et al. 2000). Poor nutrition can increase susceptibility to environmental pollutants by compromising immune function (Beck and Weinstock 1988; Rios et al. 1993). Additionally, disadvantaged neighborhoods are also exposed to greater health hazards, including tobacco and alcohol advertisements, toxic waste incinerators, and air pollution (Morello-Frosch et al. 2002). Finally, economic stress within a community may exacerbate tensions between social groups, magnify workplace stressors, and induce “maladaptive” coping behaviors, such as smoking and alcohol use (Brenner 1995). Tobacco and alcohol use can increase susceptibility to environmental toxicants that are normally metabolized by impairing host defense (Rios et al. 1993).

In general, racial minorities have lower socioeconomic position compared with whites. Although it is intuitive to hypothesize that disparities in health arise because of socioeconomic differences between racial groups, studies often find that racial disparities persist even after accounting for socioeconomics factors (Hayward et al. 2000; Sorlie et al. 1995; Williams 1999).

Although socioeconomic differences do not completely explain racial disparities, it is often argued that social class is an important mediator. That is, it is hypothesized that race determines one’s economic resources, which in turn determine health (Williams and Collins 1995). Thus, although socioeconomic conditions do not fully account for health disparities, they are a necessary part of the equation.

Neighborhood physical conditions present another structural factor that may contribute to health disparities (Cohen et al. 2003). Minorities are more likely to live in areas with building code violations and neighborhoods with deteriorated housing (Perera et al. 2002; Rosenbaum et al., unpublished data). In 1999, 3.4% of blacks, 3.8% of Hispanics, and 1.7% of Asian Americans and Pacific Islanders reported living in housing units with severe problems with heating, plumbing, electricity, public areas, or maintenance, compared with 1.5% of whites (U.S. Census Bureau 2000). Substandard housing may contribute to a variety of problems, including exposure to toxicants, increased risk of injuries from falls and fires, and illness due to ineffective waste disposal and presence of disease vectors (Bashir 2002; Jacobs et al. 2002; Krieger and Higgins 2002; Northridge et al. 2003).

Urban minorities tend to fare worse than their counterparts in rural areas (Geronimus et al. 1999; Geronimus et al. 2001). This may be due in part to land use patterns in urban areas. In Detroit, many minority neighborhoods exist next to highways that expose residents to hazards (Schulz et al. 2002). Sugrue (1996) argues that

Detroit’s highway planners were careful to ensure that construction of new … expressways would only minimally disrupt middle-class residential areas, but they had little such concern for black neighborhoods.

Similarly, New York City rezoned its neighborhoods between 1961 to 1998 so as to increase manufacturing zones in areas with higher minority populations and to decrease those zones in areas with fewer minorities (Maantay 2001). Those rezoning efforts led to a higher concentration of industrial burden within manufacturing-designated areas. Further, some policies that appear neutral prima facie may result in adverse impacts on already disadvantaged communities, as in the example of emissions trading systems and their potential to create pollution “hot spots” (Schmidt 2001; Soloman and Lee 2000).

Health infrastructure may also be associated with race. Minorities tend to reside in areas with a lower physician-per-population ratio and lower medication supply (Morrison et al. 2000; Rosenbaum et al., unpublished data; Schulz et al. 2002). Community hospitals are more likely to close in urban minority communities (Whiteis 1992). These findings suggest that segregated communities face structural disadvantages in the provision of health services.

Because so many different structural forces appear to confer disadvantage among minority communities, some scholars have suggested that they continue a history of institutionalized discrimination against minorities (Feagin and McKinney 2003; Gee 2002; Jones, 2000; Krieger et al. 1993; Massey and Denton, 1993; Squires 1994; Williams and Collins 2001). This discrimination may not have a purposeful intent but still may confer adverse impact.

### Community Stressors

Community stress theory derives from a century of research on the stress process among individuals (Aneshensel 1992; Lazarus and Folkman 1984; McEwen 1998; Selye 1936; Steptoe and Feldman 2001). “Stress” is a state of activation of physical and psychological readiness to act in order to help an organism survive external threats. “Stressors” are the factors that produce stress and include such phenomena as crime (Morenoff 2003), noise (Babisch et al. 2001; Ouis 2001), traffic (Gee and Takeuchi 2004), and litter, density, and residential crowding (Fleming et al. 1987; Evans and Lepore 1993). Stressors can result directly from environmental hazards, including technological and natural disasters (Baum et al. 1983; Brown 2002).

#### Health effects of stress.

Stressors can trigger the sympathoadrenal system, whose hallmark is rapid release of adrenalin and noradrenalin, which leads to various “fight or flight” responses, including arousal, bronchodilation, tachycardia, and increased blood pressure. The hypothalamic–pituitary–adrenal system is also activated, signified by release of corticotrophin-releasing factor, adrenocorticotropic hormone, and cortisol. These glucocorticoids have several metabolic and psychological effects, including the mobilization of energy reserves, suppression of the immune system, and heightened vigilance. Chronic activation of the stress system is believed to lead to allostatic load, which is the “wear and tear” on organ systems resulting from stress (McEwen 1998). A full discussion of the biology of stress is beyond the scope of this article but can be found in several publications (Brunner 2000; Hadley 1992; McEwen 1998).

The key point is that stressors can cause illness by weakening the body’s ability to defend against external challenges. As an example, Cohen et al. (1991) asked volunteers to self-rate their levels of stress and then randomized them to receive nasal drops containing either placebo or respiratory viruses. Rates of respiratory infection and clinically diagnosed colds followed a positive dose response with level of psychological stress. Findings from this controlled experiment were unaffected by controlling for a variety of factors (e.g., allergic status).

Intriguingly, some evidence suggests that stress may influence the internal dose of a given toxicant. This is because stress may *a*) increase the absorption of toxicants into the body through increased respiration, perspiration, and consumption (Gordon 2003); *b*) compromise host defense systems (McEwen 1998); and *c*) directly cause illness, which in turn may lead to an amplification loop whereby sick individuals are less likely to cope with environmental toxicants (Rios et al. 1993; U.S. EPA 2003b). Stress may induce or unmask a latent effect of a toxicant, possibly altering basal levels of neurofunctioning and shifting the threshold for neurotoxicity [Agency for Toxic Substances and Disease Registry (ATSDR) 1995].

Two factors are purported to determine individual response to stress: how one appraises the situation, and their general state of physical health (Lazarus and Folkman 1984; McEwen 1998). Coping resources, such as social support, help determine the extent to which a stressor is perceived as a threat and subsequent health responses (Israel et al. 2002). For example, workers with high levels of job strain and low levels of co-worker support have higher risk of cardiovascular disease than do those with similar levels of strain and more support (Johnson et al. 1996). Additionally, physical illness will impair an individual’s ability to respond to stressors. Individual stress and coping have macro-level analogs, community stressors and neighborhood resources.

#### Types of community stressors.

Community stressors can be categorized into two major types, physical and psychosocial. Physical conditions, including noise, temperature, humidity, barometric/water pressure, visible light, geomagnetism, radiation, and particulate matter, may contribute to stress (Gordon 2003). These stressors can induce a physiological response that makes the body more susceptible to illness. Heat stress, for example, induces sweating and increased skin blood flow, which in turn can facilitate the transcutaneous absorption of pesticides (Chang et al. 1994; Funckes et al. 1963; Wester et al. 1996). Individuals subject to ambient noise have higher levels of noradrenalin, a stress biomarker (Babisch et al. 2001). In a natural experiment, Evans et al. (1998) found that the chronic exposure to aircraft noise elevated resting blood pressure, norepinephrine, and epinephrine biomarker levels and decreased self-reported quality of life over a 2-year period.

Psychosocial conditions—including crowding, social disorganization, racial discrimination, fear, and economic deprivation—may also be sources of stress (Krieger and Higgins 2002; Macintyre et al. 2002). One stressor that has received extensive attention is fear of crime (Morenoff 2003; Warr and Ellison 2000). Minority neighborhoods tend to have higher crime rates, which may contribute to health disparities. Perceptions of crime and disorder within an individual’s community has been associated with numerous outcomes, including anxiety depression, posttraumatic stress disorder, and substance use (Aneshensel and Sucoff 1996; Cutrona et al. 2000; Fick and Thomas 1995; Geis and Ross 1998; Ross et al. 2000; Ross and Jang 2000). Morenoff (2003) found that the neighborhood violent crime rate was one of the “most robust” environmental predictors of infant birth weight, after controlling for both individual (e.g., smoking during pregnancy) and neighborhood (e.g. percentage of poor families) characteristics.

Physical and psychosocial stressors may interact with one another, as seen with natural and technological disasters (Ginexi et al. 2000; Kaniasty and Norris 2000). For example, the trauma of the Love Canal incident in New York resulted from both the chemical hazards and public perceptions (Edelstein and Wandersman 1987; Gibbs 1983; Holden 1980). Further, the relationship between environmental and subjective stressors occurs not only for highly salient events but also for everyday events. Gee and Takeuchi (2004), using multilevel models, reported that persons perceiving stress due to automobile traffic had greater psychological distress and lowered general health status than did those perceiving less stress. However, these outcomes were worst for persons perceiving high stress and living in high traffic areas.

#### Racial disparities in exposure to stressors.

There are racial disparities in the burden of stressors that accumulate over the life course (Geronimus et al. 2001; Holland et al. 2000; Jones 2000; Krieger et al. 1993; Williams et al. 1997). Some have called this racially differential burden of cumulative stress the “weathering hypothesis” (Astone et al. 2002; Geronimus 1996). One of the most prominent stressors may be racial discrimination (Gee 2002; Krieger and Sidney 1996; LaVeist et al. 2000; Williams and Neighbors 2001; Williams et al. 1997). Because racial discrimination has profoundly shaped the experiences of racial groups, discrimination may be among the factors that shape health disparities. Evidence suggests that racial discrimination still occurs in the present day, especially in structurally important domains such as housing, education, and employment (Essed 1992; Feagin 1991, 2000). Audit studies send a white and a minority prospective tester with identical portfolios (e.g., similar income and job titles) to assess a given housing market. These audits have consistently found that whites are favored over minorities. Hispanics, for example, are more likely to be quoted a higher rent for a given unit than are their white counterparts (Turner and Skidmore 2001). Other studies have shown that minorities are more likely to face discrimination in applying for a job (Kirschenman and Neckerman 1991) or shopping (Lee 2000).

Further, discriminatory treatment within the health care system also might contribute to disparities (Krieger 1999). Minorities appear to have longer waiting times for kidney transplants (Eggers 1995; Klassen et al. 2002) and liver transplants (Kjellstrand 1988; Young and Gaston 2000) and report less satisfaction with their medical visits (Cooper-Patrick et al. 1999; Saha et al. 2003). A review by the Institute of Medicine (2002) concluded:

Racial and ethnic minorities tend to receive a lower quality of healthcare than non-minorities, even when access-related factors, such as patients’ insurance status and income are controlled.… [T]he study committee found evidence that stereotyping, biases, and uncertainty on the part of healthcare providers can all contribute to unequal treatment.

Stress from discrimination may lead to illness. Kessler et al. (1999) have suggested that

The conjunction of high prevalence and strong impact would mean that discrimination is among the most important of all the stressful experiences that have been implicated as causes of mental health problems.

Studies have reported that stress due to racial discrimination is associated with high blood pressure (Krieger and Sidney 1996), mental health (Dion et al. 1992; Gee 2002; Kessler et al. 1999; Kuo 1976; Williams et al. 1997), and alcohol consumption (Yen et al. 1999).

### Neighborhood Resources

Although a common argument is that segregation is harmful to the health of minorities, there is some indication that segregation may have a counterbalancing effect by concentrating social resources, such as black political power (LaVeist 1993). Others have reported that the clustering of ethnic groups may build a sense of collective identity that helps mitigate trauma (Mazumdar et al. 2000). Thus, supportive social relationships within minority communities may help promote health and well-being and ameliorate the effects of community risks. Our view is that segregation concentrates both risks and resources. It is not a matter of whether segregation is either “bad” or “good,” but to what degree the negative effects of segregation outweigh positive effects.

Neighborhood resources buffer community stressors (Israel et al. 1998; Kretzman and McKnight 1993). Generally, these resources have been conceptualized in terms of relationships among residents, including social cohesion, social capital, psychological sense of community, informal social control, and community empowerment (Berkman and Clark 2003; Kawachi et al. 1999; Ross and Jang 2000; Sampson et al. 1997). “Social cohesion” is the “extent of connectedness and solidarity among groups in society” (Kawachi and Berkman 2000). Essentially, a community with a high degree of social cohesion has strong social ties between members and minimal conflict. “Social capital” can be considered a type of resource that emerges from socially cohesive groups that facilitates collective action. These resources include norms of reciprocity, aid, and interpersonal trust.

Collective efficacy, defined as “mutual trust and willingness to intervene for the common good” (Sampson et al. 1997), may mediate the adverse effects of concentrated disadvantage and fear (Ross and Jang 2000). Pastor et al. (2001) suggested that social capital was stronger in communities with less “ethnic churning,” referring to the replacement of one minority group with another within a community. They argued that ethnic churning may “weaken the usual social bonds constituted by race and make an area more susceptible to siting of noxious land uses.” Their data indicated that ethnic churning in Los Angeles was associated with the siting of hazardous waste storage and disposal facilities over a two-decade period, after adjusting for economic factors.

Another potential resource is residents’ ability to control their environment, which may mitigate community problems in two ways. First, empowered communities may be able to protect themselves from the instruction of new hazards and eliminate extant ones (Bullard and Wright 1993; Lee 1993; Morello-Frosch et al. 2002; Phoenix 1993; Rich et al. 1995; Zimmerman 2000). These communities may also be able to control the political arena that shapes their health beyond the effect of environmental pollutants. Black political participation, defined by the presence of African-American legislators, has been associated with lower mortality rates in African-American communities (LaVeist 1993). This is possibly due to a higher preponderance among African-American communities to provide a wider range of social services compared with white communities (Schneider and Logan 1982). Second, control per se may be an important factor determining stress and health. Workers with greater control over their work process have lower risk of cardiovascular disease than do workers with less control (Karasek and Theorell 1990; Kuper and Marmot 2003; Landsbergis et al. 1997). Further, collective control by workers and their unions may also provide health benefits (Johnson 1989; Sorensen et al. 2004).

### Community Stress

The cumulation of environmental pollutants, structural process, community stressors, and neighborhood resources is community stress. Community stress is a state of ecological vulnerability. Community resources help buffer community stressors and protect against environmental exposure, but when resources are inadequate, community stress arises. Structural factors constrain the limits of resources and stressors.

Although several factors cross the threshold from “community” to “individual,” we focus on the intersection between community stress and individual stress. In particular, community stress may itself lead to individual stressors. These individual stressors may in turn lead to individual stress and subsequent illness. The terrorist attacks of 11 September 2001 provide an extreme example of how community stress can translate to individuals. The attack was a threat to the American “community.” Although most citizens were not close to the epicenter, many individuals across the United States felt some measure of distress from the attack (Schlenger et al. 2002; Schuster et al. 2001).

## Future Directions

Our stress–exposure–disease framework is meant to stimulate dialogue between environmental and social scientists. Several avenues for future work are suggested. First and foremost, although several components within the framework have undergone extensive study, such as between individual stress and health, relatively little work has attempted to integrate the elements as a whole. Studies are just beginning to consider the connections among factors at multiple levels, such as among community stress, individual stress, and health. Future work should continue to test the components of the framework and incorporate multilevel modeling (Raudenbush and Bryk 2002). Longitudinal studies will be necessary to establish the temporal ordering between variables.

Second, public health should more seriously consider the role that residential segregation plays in the production of health disparities. Several lines of inquiry are possible regarding segregation alone. For example, what role might environmental risk perception play in maintaining segregation? Are certain dimensions of segregation more important than others? Are the mechanisms linking segregation to health all negative, or might there be some health-promoting pathways, such as in the clustering of cultural resources? What are the forms of segregation outside of the United States, and are the mechanisms similar? Does the relationship between segregation and health generalize to all ethnic groups?

Third, we hope that this framework will encourage the environmental justice movement to expand the notion of “environmental hazards” to include community stressors. Are minority communities more likely to receive the siting of workplaces with high job strain (Karasek and Theorell 1990)? Do differences in community stress lead to the “weathering” (Geronimus 1996) of minority communities compared with whites? This means not only examining the main effects of stress and toxicants, but also examining whether psychosocial stress may potentiate (i.e., amplify) the effects of toxicants on the body.

Fourth, research should not only examine the relationship between minority communities and exposures, but also study how the structural conditions of communities may confer additional vulnerability. Disadvantaged communities may be more vulnerable to exposure to environmental hazards because structural conditions, such as substandard housing, may render them more likely to be exposed than are counterparts in more advantaged communities equally distant from these hazards. That is, do minority communities have less protection against a given level of exposure, and do these disparities in protection result from differential social policy?

## Conclusions

Our work has implications for environmental justice by suggesting that exposure to physical and chemical hazards is only one route whereby neighborhoods affect the health of racial minorities. Health promotion may require policies and interventions aimed at eliminating environmental toxicants, fostering community resources, and reducing social stressors. Reduction of the gap in health between advantaged and disadvantaged groups, however, may require interventions targeted at eliminating the gap in advantages themselves.

We emphasize racial differences in exposure to stress, rather than racial differences in response to stress. The former conceptualization emphasizes interventions on macro-level social policy (e.g., housing policy), whereas the latter perspective emphasizes interventions at the micro level (e.g., psychological counseling or pharmacological agents). Although micro-level approaches are useful, one disadvantage is that individual interventions require tremendous resources in order to manifest outcomes at the population level (and hence reduce group differences) and, further, are less efficient because interventions must be reapplied to each new birth cohort. However, policy-level changes that target socially produced stressors may prove a promising way to improve the public’s health.

## Figures and Tables

**Table 1:** Segregation of ethnic minorities compared with whites, United States, 1980–2000.

	1980	1990	2000
Native Americans	37.3	36.8	33.3
African Americans	72.7	67.8	64.0
Asian Americans and Pacific Islanders	40.5	41.2	41.1
Hispanics	50.2	50.0	50.9

Segregation was determined using the index of dissimilarity, which measures the evenness of groups over space and can be interpreted as the percentage of a particular group who would have to move in order integrate the two groups over the region as a whole. For example, in the year 2000, 64% of all African Americans (or whites) would have to move to another census tract in order to integrate all metropolitan areas nationwide. Data are adapted from the U.S. Census Bureau (2003).
